# Supplementary material for: Population Status and Vulnerability of Mantidactylus pauliani from Ankaratra Protected Area, Madagascar
Source: Animals (Basel). 2023 Aug 25;13(17):2706. doi: 10.3390/ani13172706 (PMC10486469; doi:10.3390/ani13172706)
Supplement: Supplementary file 1 [file animals-13-02706-s001.zip › animals-2479357-supplementary.pdf]

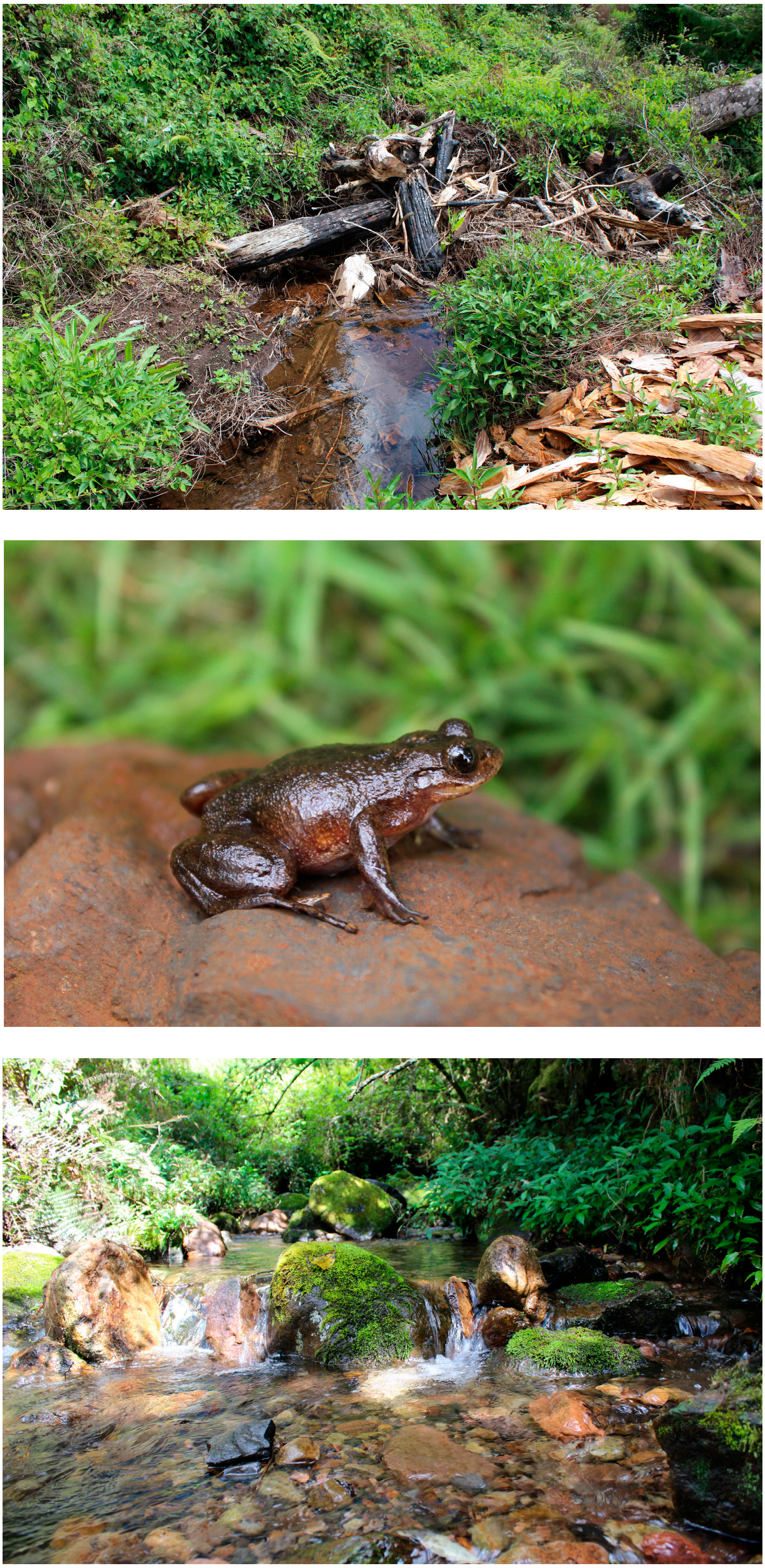

Figure S1. Photo of pressure and threats in Ankaratra Massif (top image). Photo of *Mantidactylus pauliani* (middle image) and its habitat (bottom image).
